# Draft Genome Sequence of Pseudomonas gingeri Strain LMG 5327, the Causative Agent of Ginger Blotch in Agaricus bisporus

**DOI:** 10.1128/genomeA.00196-18

**Published:** 2018-03-29

**Authors:** Tahereh Bahrami, Samira Zarvandi, René De Mot, Harald Gross, Majid Changi-Ashtiani, Tina Shahani, Hassan Rokni-Zadeh

**Affiliations:** a Department of Medical Biotechnology and Nanotechnology, School of Medicine, Zanjan University of Medical Sciences, Zanjan, Iran; b Department of Microbial and Molecular Systems, Centre of Microbial and Plant Genetics, KU Leuven, Heverlee, Belgium; c Department of Pharmaceutical Biology, Pharmaceutical Institute, University of Tübingen, Tübingen, Germany; d School of Mathematics, Institute for Research in Fundamental Sciences (IPM), Tehran, Iran; e Department of Genetics and Molecular Medicine, School of Medicine, Zanjan University of Medical Sciences, Zanjan, Iran; f School of Biological Sciences, Institute for Fundamental Sciences (IPM), Tehran, Iran

## Abstract

The draft genome sequence of Pseudomonas gingeri LMG 5327 (NCPPB 3146), the causative agent of ginger blotch in Agaricus bisporus, is reported. Together with another mushroom pathogen, Pseudomonas agarici, it belongs to a distinct phylogenomic group.

## GENOME ANNOUNCEMENT

Pseudomonas gingeri is responsible for ginger blotch disease on Agaricus bisporus fruit bodies (1–3). This species is related to another mushroom pathogen, P. agarici (4, 5), that belongs to a distinct phylogenomic group (6, 7). Here, we report the draft genome sequence of Pseudomonas gingeri type strain LMG 5327 (NCPPB 3146), determined by an Illumina HiSeq 2000 sequencing system. A total of 9,604,938 reads were used for *de novo* assembly with the Velvet assembler (8). A total of 192 contigs, with an *N*_50_ value of 69,954 bp (about 120-fold median coverage), were generated. The final assembled length comprises 7,643,850 bp, with a G+C content of 62.6%, and the longest contig size is 252,545 bp. Automated annotation using the NCBI Prokaryotic Genome Annotation Pipeline (PGAP) (9) predicted 6,860 coding DNA sequences (CDS) and 57 tRNA genes.

The characteristic secretion systems of proteobacteria are all present in P. gingeri, including gene clusters of type III and type VI secretion genes, with potential relevance for its pathogenicity and interaction with competing bacteria, respectively. In the *mutS-cinA* intergenic region, a tailocin gene cluster has been recruited for the production of two phage tail-like bacteriocins of different phage ancestries (Siphoviridae and Myoviridae) to support interference competition (10). The capacity to produce specialized metabolites that mediate microbial antagonism is inferred from the presence of biosynthetic gene clusters for hydrogen cyanide and 2,4-diacetylphloroglucinol (11). Besides the nonribosomal peptide synthetases for pyoverdine synthesis, a number of gene clusters with such enzymes are present, which likely participate in building the peptide-based metabolome of P. gingeri. The presence of a *luxR-luxI* gene pair can be linked to the high-level production of 3-oxo-C_4_-homoserine lactone (12). Adding to the anabolic capacity is a gene cluster for degradation of alkyl-substituted phenols, shared with Pseudomonas alkylphenolica (13), and detoxification of organoarsenicals (14).

The draft genome sequence of P. gingeri LMG 5327 reported here is a valuable source of information for studying the bacterium’s interaction with its host and its pathogenicity.

### Accession number(s).

This whole-genome shotgun project has been deposited at DDBJ/ENA/GenBank under the accession number POWE00000000. The version described in this paper is version POWE01000000.
